# Primary Disseminated Multidrug-Resistant Tuberculosis of the Lungs, Brain, Meninges, and Abdomen: The World’s First Case

**DOI:** 10.7759/cureus.41302

**Published:** 2023-07-03

**Authors:** Sankalp Yadav

**Affiliations:** 1 Medicine, Shri Madan Lal Khurana Chest Clinic, Moti Nagar, New Delhi, IND

**Keywords:** intracranial tuberculoma, meningitis, mtb (mycobacterium tuberculosis), multidrug-resistant tuberculosis, disseminated tuberculosis

## Abstract

Tuberculosis is a highly infectious disease. It usually infects the lung, but dissemination to different organs results in a severe form of tuberculosis, i.e., disseminated tuberculosis. The situation becomes even more challenging when the infection is due to multidrug-resistant strains of *Mycobacterium tuberculosis*. The present case is a very rare one where a 17-year-old Indian girl presented with headache, vomiting, cough with expectoration, abdominal pain, and a seizure. A diagnostic workup led to the diagnosis of primary disseminated multidrug-resistant tuberculosis of the lungs, brain, meninges, and abdomen. She has been prescribed an anti-tubercular regimen per the national guidelines.

## Introduction

Tuberculosis is a disease due to infection by *Mycobacterium tuberculosis* [1]. It is the second-highest contributor to morbidity and mortality after coronavirus disease 2019 (COVID-19) [2]. According to the latest World Health Organization (WHO) Global Tuberculosis Report, about 21.4 lakh cases of tuberculosis were notified in the year 2021, which was 18% more as compared to the previous year [3]. Often, the presentations are pulmonary due to the entry of the bacteria through inhalation, but reports of primary extrapulmonary involvement are also available in the medical literature [4].

Disseminated tuberculosis is defined as the simultaneous involvement of no less than two or more nonconsecutive organs or tubercular involvement of the blood or bone marrow [5]. Often, the primary focus is on the lungs, which spread by hematogenous mode to other organs [5]. This form of tuberculosis is severe and requires a detailed diagnostic workup and longer treatment [5,6].

Drug resistance is rising in tuberculosis. In the year 2021, about 70787 cases of various forms of drug-resistant tuberculosis were diagnosed in India [7]. Multidrug-resistant tuberculosis refers to resistance to the two most potent first-line anti-tubercular drugs, i.e., rifampicin and isoniazid [7]. This type of tuberculosis is a result of spontaneous mutations in the genes of the bacilli and it is vital to have a very high load of bacteria, i.e., *Mycobacterium tuberculosis*, for the development of primary multidrug-resistant tuberculosis [8]. It is a man-made problem and is mainly due to improper or poorly administered treatment [4]. According to the recent data of the Government of India in March 2021, the approximate number of multidrug-resistant/rifampicin mono-resistant cases in India was 124000 (9.1 per 0.1 million population) [9].

Primary multidrug-resistant tuberculosis is a condition where there is no prior tuberculosis treatment history or a history of treatment for less than one month [10]. The prevalence of primary multidrug-resistant tuberculosis is nearly 3% [10].

The present case is a very rare one where a young Indian girl presented with a headache, fever, vomiting, cough with expectoration, abdominal pain, and a seizure. A diagnostic workup led to the diagnosis of primary disseminated multidrug-resistant tuberculosis of the lungs, brain, meninges, and abdomen. She was prescribed anti-tubercular chemotherapy according to the national guidelines.

## Case presentation

In the year 2018, a 17-year-old non-diabetic Indian female came with complaints of headache, vomiting, fever, cough with expectoration, seizure, and abdominal pain for 20 days. She was asymptomatic 20 days ago when she developed a severe headache that was constant, located on the left side, and was relieved only for a short time after taking acetaminophen sulfate. This was associated with three episodes of non-projectile vomiting. She also had a fever for the last 18 days; initially, it was once every two days, but it was daily for the last week. She had a nocturnal fever and was associated with night sweats; however, there were no chills or rigors. Further, she also complained of a cough with yellow-colored, non-foul-smelling expectoration for two weeks. For the last week, she also had abdominal pain that was on and off and was not associated with any aggravating or relieving factors. Lastly, she had two episodes of seizures in the last three days. It was associated with neck pain and left eyelid ptosis.

Furthermore, there was a negative history of trauma, hemoptysis, or remarkable weight loss. Also, there was no history of tuberculosis in her or any of her close contacts, and there was no other history of any major surgical or medical illness. She was fully vaccinated for her age.

On examination, she was drowsy, cooperative, and well-oriented to time, place, and person. Her vitals were a pulse of 99 per minute, a blood pressure of 110/70 mm of Hg, a respiratory rate of 21 breaths per minute, oxygen saturation (SpO_2_) of 96% in room air, and a temperature of 99 degrees Celsius. On auscultation, there was crepitation on the bilateral upper lobes of the lungs. The abdominal examination revealed tenderness in the right lumbar, epigastric, and umbilical regions. However, there was no shifting dullness or dilated veins. The rest of the systemic examination was within normal limits. A neurological examination revealed a drowsy patient with bilateral ptosis (left more than right). The motor examination was remarkable for power (upper and lower limbs) 3/5. Neck rigidity was present with positive Kernig and Brudzinski's signs. Fundoscopy was unremarkable. Moreover, there was no cervical, supraclavicular, or axillary lymphadenopathy, jaundice, clubbing, pretibial edema, pallor, or cyanosis.

Based on the clinical features and endemic background, a diagnosis of probable tuberculosis was made. She was advised to undergo sputum microscopy (Ziehl-Neelsen staining for acid-fast bacilli), a chest radiograph, a cartridge-based nucleic acid amplification test (CBNAAT) of the sputum, and an ultrasound of the whole abdomen with other routine investigations.

**Figure 1:**
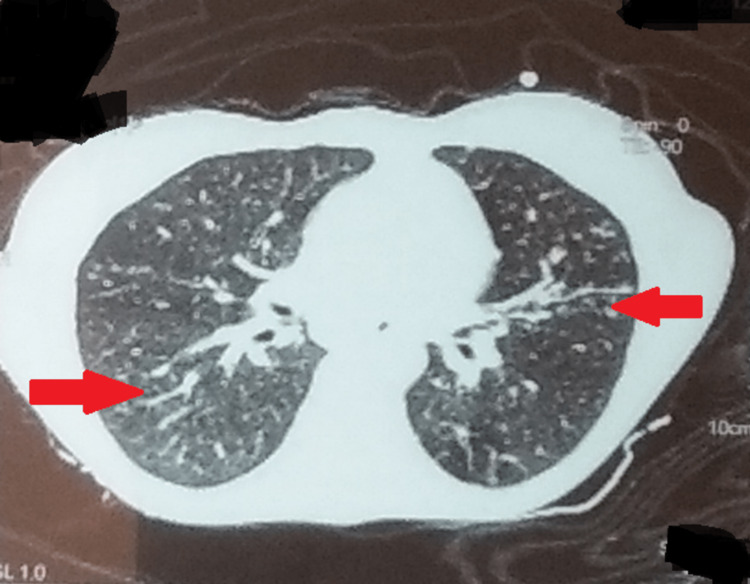
CECT chest showing bilateral involvement of the lungs CECT: contrast-enhanced computed tomography

Results confirmed the initial diagnosis with a low detection of *Mycobacterium tuberculosis *on CBNAAT with resistance to rifampicin, but sputum microscopy was negative. Additionally, one more sample was sent for the line probe assay and culture for drug susceptibility testing. Her chest radiograph was suggestive of small consolidation in the bilateral upper lobes and right middle lobe. An ultrasound of the whole abdomen was remarkable for several necrotic lymph nodes in the pre- and para-aortic stations. Further, a contrast-enhanced computed tomography (CT) of the chest revealed multiple small patchy areas of consolidation with an air bronchogram in both the upper lobes and the right middle lobe (Figure 1). Multiple necrotic mediastinal lymph nodes were seen in the right upper, paratracheal, bilateral hilar, carinal, and subcarinal stations.

**Figure 2:**
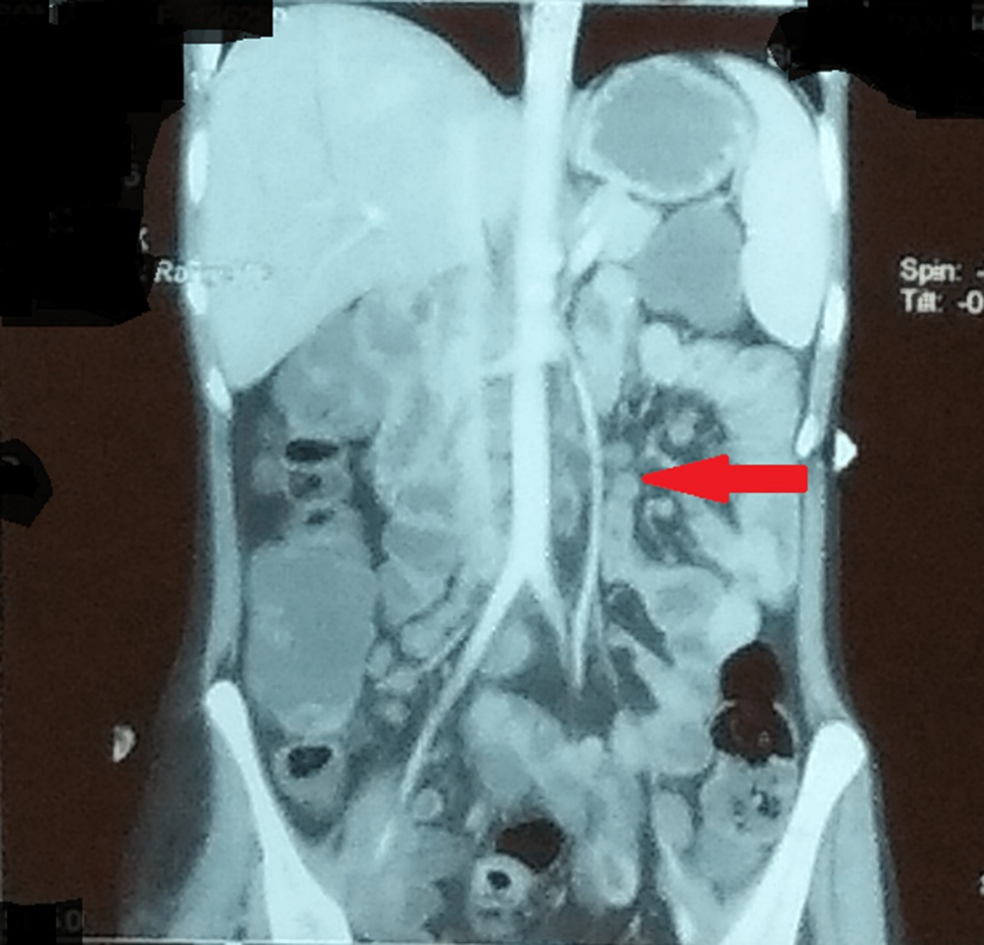
CECT whole abdomen showing multiple enlarged necrotic lymph nodes in the pre- and para-aortic, inter-aorto-caval stations CECT: contrast-enhanced computed tomography

**Figure 3:**
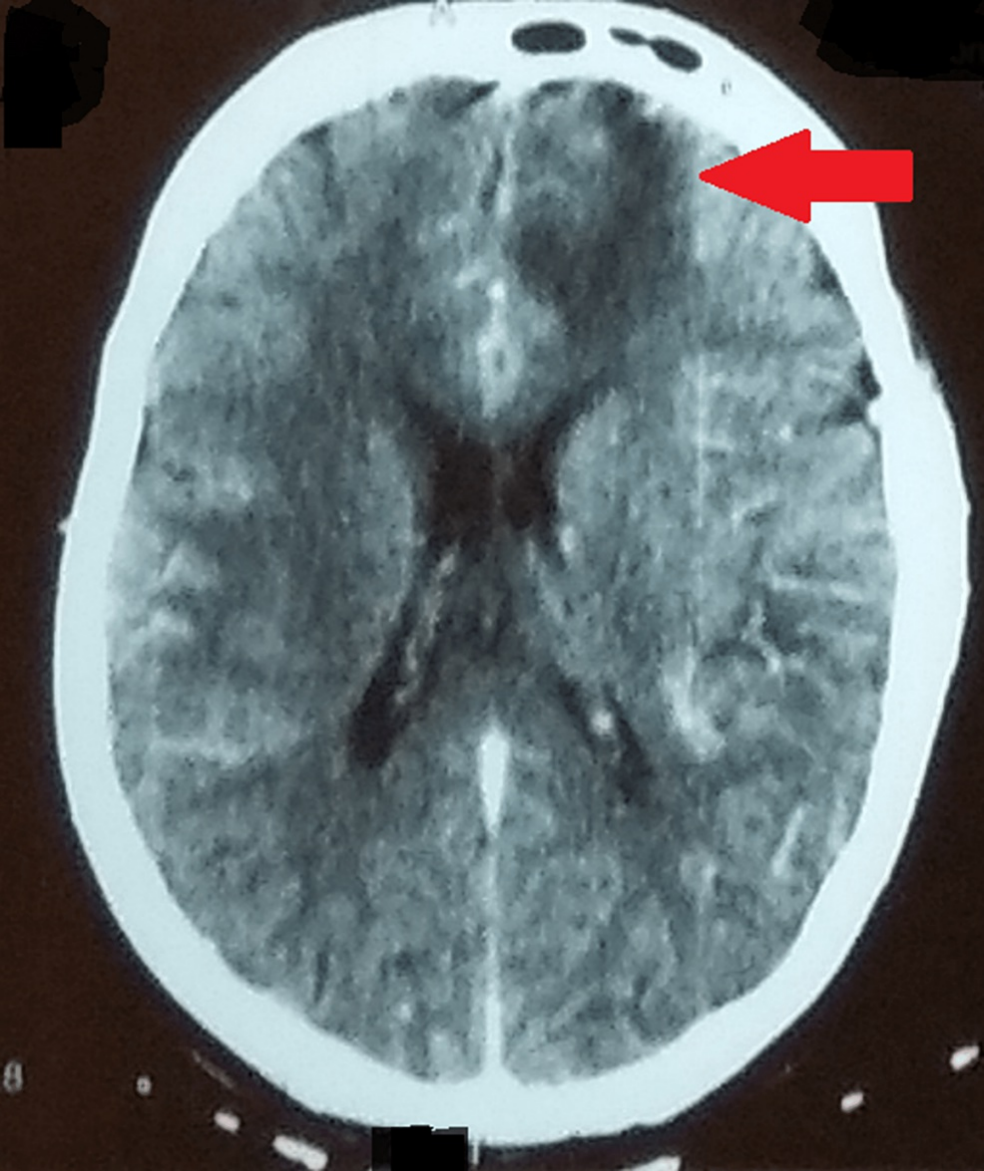
Plain CT brain showing an ill-defined isodense lesion in the left basifrontal region CT: computed tomography

**Figure 4:**
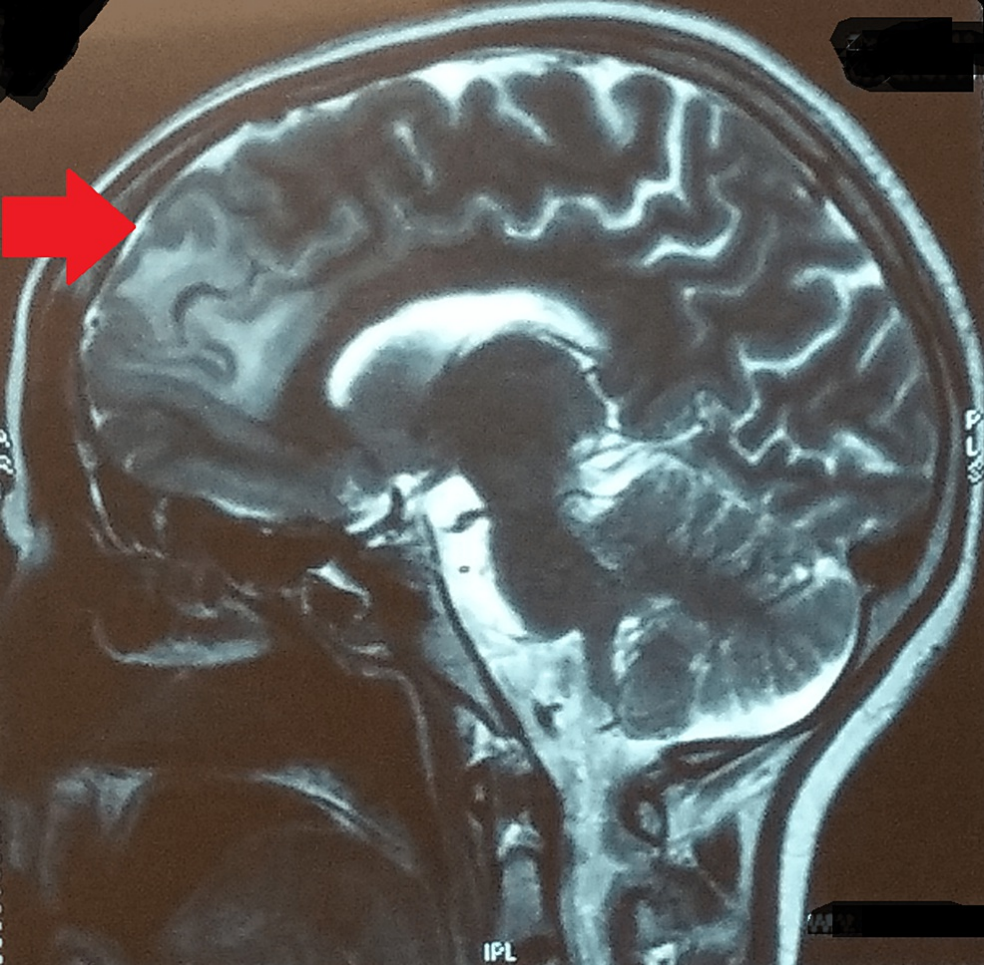
MRI brain showing left parasagittal frontal lobe hyperintensity MRI: magnetic resonance imaging

**Figure 5:**
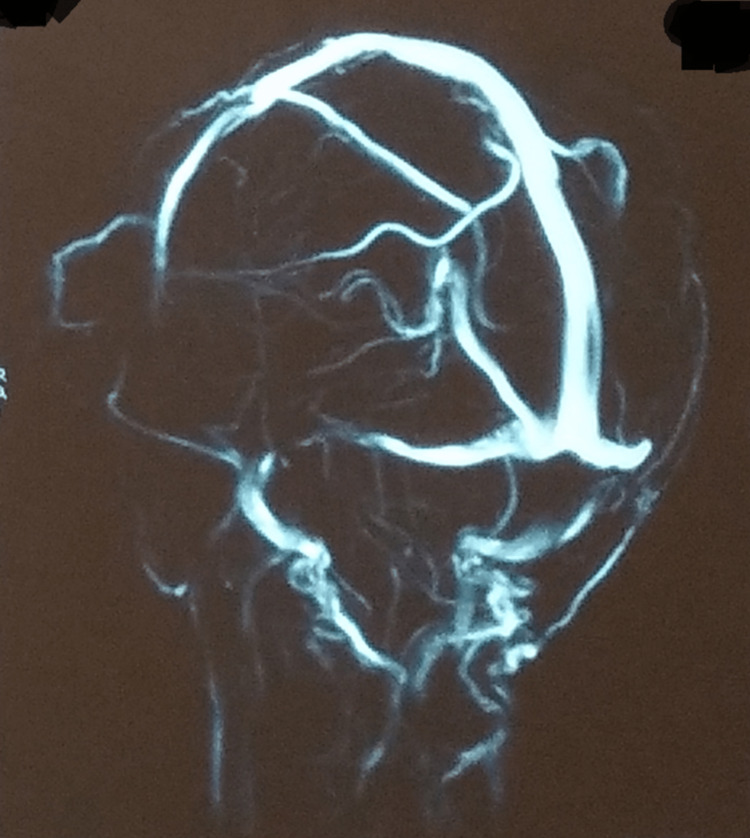
A normal MRA brain showing no infarcts MRA: magnetic resonance angiography

A contrast-enhanced CT of the whole abdomen was remarkable for multiple enlarged necrotic lymph nodes in the pre- and para-aortic, inter-aorto-caval stations, with the largest measuring about 20 X 11 mm (Figure 2). A plain CT of the brain revealed an ill-defined isodense lesion measuring about 2.1 X 1.8 cm in the left basifrontal region, extending into the parasagittal region. Mild edema was noted around the lesion. Hypodensity was seen in the entire right external capsule region (Figure 3). On magnetic resonance imaging (MRI) of the brain with contrast, a left parasagittal frontal lobe hyperintensity intending in the white matter with blurring of cornices was seen (Figure 4). Inter-hemispheric/sural space enhancement was noted, suggesting a space-occupying lesion. Blurring of the adjacent parasagittal right frontal cortex was seen. Besides, there were no infarcts seen on magnetic resonance angiography or venography (Figure 5). The reports of the line probe assay and culture of sputum confirmed the detection of *Mycobacterium tuberculosis* with resistance to rifampicin and isoniazid. And a lumbar puncture revealed turbid cerebrospinal fluid with an increased white blood cell count and protein levels and a low sugar level suggestive of bacterial meningitis.

Finally, a diagnosis of primary disseminated multidrug-resistant tuberculosis of the lungs, brain, meninges, and abdomen was made, and she was advised to undergo a detailed pretreatment evaluation per the national guidelines (Table 1) [11].

**Table 1:** Pretreatment evaluation per the national guidelines

Test	Result	Reference range
Hemoglobin	11 g/dL	11.9-15
Platelet count	3.4 x 10^9^/L	1.5-4.0 x 10^9^
Total leukocyte count	9.2 × 10^9^/L	4-10
Erythrocyte Sedimentation Rate	80 mm/hr	0-20
Bilirubin (conjugated)	0.7 µmol/L	<1
Human immunodeficiency virus	Non-reactive	Reactive-Non-reactive
Fasting blood sugar	4.14 mmol/L	3.9-5.6
Serum creatinine	57 µmol/L	53-97.2
Urine routine and microscopic	Normal	Normal-Abnormal
Urine pregnancy test	Negative	Negative-Positive
Audiogram	Hearing loss is absent	Hearing loss is present-Absent
Serum thyroid-stimulating hormone levels	0.6 mU/L	0.4-4.0
Mental health assessment	Absent	Psychiatric issues are present-Absent
Surgical evaluation (Lung resection surgery)	Not indicated	Indicated-Not indicated

As her pretreatment evaluation was normal, she was initiated on a conventional multidrug-resistant tuberculosis regimen per the national guidelines (Table 2) [11]. Because of suspected optic atrophy, ethambutol was substituted by pyrazinamide by the drug-resistant tuberculosis committee at the nodal drug-resistant tuberculosis center. Besides, she has also been prescribed a tablet of levetiracetam (1 gm twice a day), a tablet of pyridoxine (20 mg once a day), a tablet of prednisolone (40 mg once a day), and a tablet of pantoprazole (40 mg before breakfast). She responded well to her treatment initially with no adverse drug reactions and there was recovery in bilateral ophthalmoplegia. She was discharged (after 14 days) in stable condition with advice to follow up in the outpatient department, but she was lost to follow up as her parents migrated to a different state.

**Table 2:** Conventional multidrug-resistant tuberculosis regimen according to the national guidelines

Drug	Dose	Route of administration	Duration
Tablet Pyrazinamide	1250 mg	Per oral	Eighteen months
Tablet Levofloxacin	750 mg	Per oral	Eighteen months
Tablet Cycloserine	500 mg	Per oral	Eighteen months
Tablet Ethionamide	500 mg	Per oral	Eighteen months
Injection Kanamycin	750 mg	Intramuscular	Six months

## Discussion

Multidrug-resistant tuberculosis is a considerable threat to the public health [12]. The disease is often reported late, especially when organs other than the lungs are involved, and this adversely affects the management and treatment outcomes [12]. The management of multidrug-resistant tuberculosis is complex and associated with a longer duration of treatment, reduced patient compliance, a high pill burden, and greater chances of adverse drug reactions [12].

Disseminated multidrug-resistant tuberculosis is a challenging clinical condition [13]. There is a higher probability of developing disseminated tuberculosis in immunocompromised patients; however, it can rarely manifest in immunocompetent cases [14]. A higher incidence of disseminated and pulmonary tuberculosis is seen in men [14]. Usually, it occurs due to the reactivation of an old latent bacterial infection, but it is extremely fatal if left untreated [14]. Some of the strongest mortality predictors are age, delay in presentation, and serious underlying disease [14].

The WHO recommends the use of a multi-drug regimen for this type of tuberculosis [11]. However, there are no clinical guidelines for a definite period for which the treatment should be continued, especially in cases of extrapulmonary involvement.

Tubercular engagement of the central nervous system is noted in about 1.5% of total cases of tuberculosis and is predominantly seen as meningitis, while other presentations include tuberculoma and spinal arachnoiditis [14].

In the management of tuberculosis of the central nervous system, drugs like ethionamide, cycloserine, prothionamide, and fluoroquinolones such as moxifloxacin (high dose) are commonly used due to their significant penetration beyond the blood-brain-barrier [12].

Based on the available literature, largely from case reports and a case series, it is evident that multidrug-resistant tuberculosis with involvement of the meninges has a poor prognosis [12]. Further studies from the United States and South Africa on 26 and 30 multidrug-resistant tubercular meningitis cases reported 73% and 66.7% mortality, respectively [12]. In general, the mortality rates in tuberculous meningitis vary from 55 to 75% [14].

Cases of disseminated multidrug-resistant tuberculosis are available in the literature. However, no similar case with no history of tuberculosis and with involvement of the lungs, brain, meninges, and abdomen in an immunocompetent host has ever been reported. This emphasizes the importance of a detailed diagnostic workup, especially in endemic settings.

In endemic countries, all patients diagnosed with tuberculosis must undergo universal drug susceptibility testing to determine any drug resistance [11]. National Programs should be made patient-friendly to reduce the time lag from the first visit to treatment initiation. Nevertheless, this was only one case, and thus it is essential that large-scale data similar to this case be reported, especially from endemic countries.

## Conclusions

Multidrug-resistant tuberculosis of the lungs, brain, meninges, and abdomen is an infrequent condition. It requires a high index of suspicion for timely diagnosis and a strong diagnostic workup backed by exhaustive management with second-line anti-tubercular drugs. It is imperative that primary care physicians be trained regularly to avoid unnecessary delays in management, which could prove fatal.
